# Geometric Evolvement, Simulation, and Test of a Bionic Lateral PDC Reamer Bit Inspired by *Capra sibirica* Horn

**DOI:** 10.1155/2022/4470153

**Published:** 2022-02-17

**Authors:** Chaoyu Chen, Jialin Yan, Yuntian Zou, Haifeng Luo

**Affiliations:** ^1^School of Technology, Beijing Forestry University, Beijing, China; ^2^Key Lab of State Forestry Administration on Forestry Equipment and Automation, China; ^3^Research Institute for Frontier Science, Beihang University, Beijing, China

## Abstract

PDC (polycrystalline diamond compact) bit is the key equipment for drilling holes inside the rock in oil and mining industry. Inspired by the shape and structure of *Capra sibirica* horn, a bionic lateral PDC reamer bit with variable lateral reaming radius was developed. Side view of *Capra sibirica* horn was employed for fitting the horn shape curve based on picture processing method. PDC teeth were arranged on the horn shape blade imitating the transverse ridges on the horn to cut the rock material, found with only 30% utilization rate of the total teeth and load concentration of the last tooth. A bionic blade curve evolved from the *Capra sibirica* horn was defined with geometric method for the lateral reamer bit; the utilization rate of the teeth on the bionic blade curve was improved to 90% with uniformly distributed reaming load. Multigroup simulations were conducted with the finite element method; the effects of bit revolution speed and rotation feed speed of the bionic blade to reaming load were emphatically studied. Concrete sample was reamed indoors from 240 mm to 407 mm in diameter, and the bionic lateral PDC reamer bit was approved by the test result.

## 1. Introduction

Drilling is an important procedure in oil and mining industry; the hole inside the rocks is drilled mostly by the PDC bit, which has a market share of about 85% [1]. The PDC bit for drilling is often constructed with several blades, and the PDC teeth (hereinafter referred to as tooth and teeth) are welded along the edge of the blades in a manner that all the teeth can cut the rock material one by one when the bit rotates and feeds forward. The shape and diameter of the holes drilled inside the rock are directly determined by the bit blades and teeth on them. The existing blades of the PDC bit are mostly fixed structure. The hole is thus a single straight cylinder shape with constant diameter, which is equal to the cutting dimension of the bit teeth [2–4]. The hole in rock can be reamed by another bigger size bit, which is installed behind the guiding bit in the axial feeding direction of hole [5, 6]. The nowadays PDC bits are not competent in laterally reaming the hole due to the structure restriction of the fixed blades. A type of drill bit for foundation stake in the civil engineering of rocky area is constructed with a metamorphic triangle blade; the hole with a cone shape for the stake can be reamed laterally by the teeth on the hypotenuse of triangle blades [7]. The PDC bit with metamorphic blades thus variably cutting position of the teeth is feasible to expand the research and application of the drilling bit.

Bionic design for mechanical engineering, for instance the cutting tools, is an effective method in special applications and has been studied since the bionic design concept was proposed [8, 9]. Meyers et al. focused on sharp edges and serrations as important survival and predating mechanisms in a number of plants, insects, fishes, and mammals; some bioinspired applications also were studied [10]. Tian et al. designed a bionic blade by extracting the cutting tooth profile curve of the *B. horsfieldi* palate; the blade has better cutting force and energy consumption reduction performance than ordinary blades [11]. Du et al. designed a biomimetic cutter to reduce the force and energy required to cut the stalks of tea plants inspired by the special geometrical structure of the teeth of crickets [12]. Tang et al. designed a self-adaptive bionic PDC bit inspired by cat claw to solve the problem of short service life and low comprehensive drilling efficiency of PDC bits [13]. *Capra sibirica* is the wildlife conservation at the national level. It is distributed in Xinjiang, Inner Mongolia of China, and central Asia. Inspired by the shape of horn and transverse ridges structure on it, a bionic blade for the lateral PDC reamer bit was proposed obeying the basic PDC bit technology [14]. The bionic blade was evolved from the horn shape and redefined to obtain a variable reaming radius and also to improve the utilization rate of the teeth, thus decentralizing the reaming load to most teeth on the bit. The reaming load of the bionic lateral PDC reamer bit were studied with the finite element method and further with a prototype and concrete samples.

## 2. Materials and Methods

### 2.1. Materials

**Figure 1:**
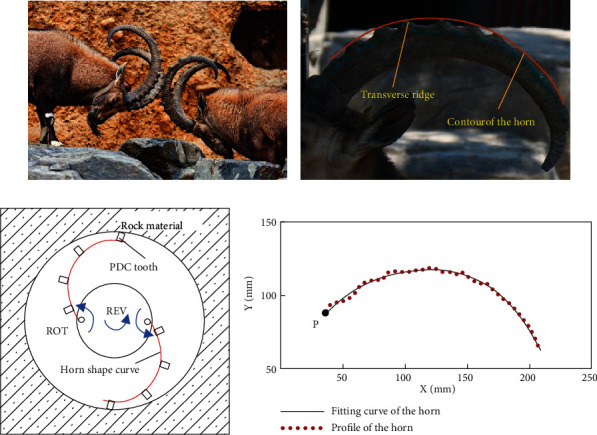
(a) Picture of *Capra sibirica*. (b) Sideview image of horn. (c) Bionic reaming method. (d) Profile and fitting curve of the horn.

The horn on the larvae of male *Capra sibirica* is short and straight; with the grown up of larvae, their horn will bend year by year. The horn on the adult individual is a scimitar shape, which is used to fight and win the spouse. As shown in Figure 1(a), in the fight of two male *Capra sibirica*, the horn is rotated forward by head to control the attack distance. The transverse ridges on the horn will contact and increase the contact friction between them, thus preventing excessive injuries in fight [15, 16], as the side view picture shown in Figure 1(b).

The shape and structure of the *Capra sibirica* horn can be used for reference in the PDC bit design. The lateral reaming radius of the PDC bit can thus be variable with a rotational horn shape blade, which has a changeable attacking distance, as shown in Figure 1(c). The PDC teeth can be arranged on the horn shape blade, contact, and cut the material as the horn shape blade is rotated outward. The average length of the *Capra sibirica* horn is about 1000 to 1500 mm [16]. In order to get the curve of the horn, side view of the *Capra sibirica* horn in Figure 1(b) has been used for extracting the profile and further fitting a horn shape blade curve based on image processing technology [17, 18]. The extracted profile and fitting curve of the *Capra sibirica* horn is shown in Figure 1(d). According to an experimental reaming radius (from 100 mm to 200 mm), the extracted profile and fitting curve of the horn has been reduced to 15% of its original size, as shown in Figure 1(d). The four-order polynomial fitting function of the horn curve based the least square approach [19] is obtained as:

(1) $$\begin{array}{c} y=-2.4\times 10^{-7}x^{4}+1.01\times 10^{-4}x^{3}-0.0194x^{2}+1.96x+36.1, \end{array}$$

where 36.6 mm ≤ *x* ≤ 208.7mm. And the beginning point of the horn shape blade is marked with *P*, as shown in Figure 1(d).

## 3. Methods

### 3.1. Horn Shape Blade

The equally distributed transverse ridges on the *Capra sibirica* horn can help them fighting with controlled attacking distances; the PDC teeth are thus similarly spread averagely on the horn shape blade to cut the material in sequence from the *P* point to its distal end. The horn shape blade for the lateral PDC reamer bit was proposed to work in the following method.

**Figure 2:**
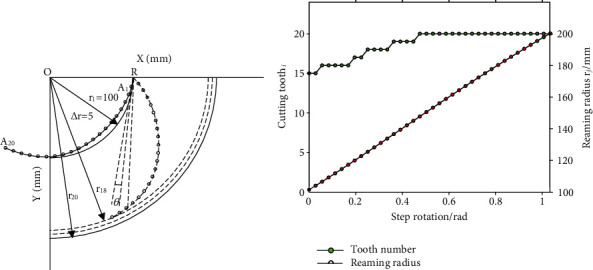
Curve of the horn shape blade. (a) Horn shape blade curve. (b) Reaming radius and related tooth.

20 points from *A*_1_ to *A*_20_ are spread averagely on the horn shape blade curve (shown in Figures 1(d) and 2(a)). Each point *A*_*i*_(*x*_*i*_, *y*_*i*_)(*i* = 1, 2, 3, ⋯, 20) is arranged with a PDC tooth for cutting the material. The coordinate of tooth *A*_*i*_ on the horn shape curve can be obtained with Equation (1) as:

(2) $$\begin{array}{c} \left\{\begin{array}{c} \int_{36.6}^{x_{i}}\sqrt{{\left(\frac{dy}{dx}\right)}^{2}+1}dx=\frac{i}{20}\int_{36.6}^{208.8}\sqrt{{\left(\frac{dy}{dx}\right)}^{2}+1}dx,\\ y_{i}=-2.4\times 10^{-7}x_{i}^{4}+1.01\times 10^{-4}x_{i}^{3}-0.0194x_{i}^{2}+1.96x_{i}+36.1, \end{array}\right.\left(i=1,2,\cdots ,20\right). \end{array}$$

Move the horn shape blade curve until the beginning point *P* of it (shown in Figure 1(d)) coinciding with the rotation joint point *R* (shown in Figure 2(a)) on the bit body. Rotate the horn shape blade curve until it is tangent with the initial reaming arc with radius of *r*_1_(*r*_1_ = 100mm), as the solid curve RA_20_ and *r*_1_ shown in Figure 2(a). The initial position of each point *A*_*i*_ on the curve RA_20_ is further obtained with Equation (2) as:

(3) $$\begin{array}{c} \left\{\begin{array}{c} x_{i}^{\prime }=\left(x_{i}-36.6\right)\cdot \cos\left(\frac{‐143\cdot \pi }{180}\right)-\left(y_{i}-86.3\right)\cdot \sin\left(\frac{‐143\cdot \pi }{180}\right)+95,\\ y_{i}^{\prime }=\left(x_{i}-36.6\right)\cdot \sin\left(\frac{‐143\cdot \pi }{180}\right)+\left(y_{i}-86.3\right)\cdot \cos\left(\frac{‐143\cdot \pi }{180}\right), \end{array}\right.\left(i=1,2,3,\cdots ,20\right). \end{array}$$

The horn shape blade curve RA_20_ is unfolded step by step (*j* is the step label) with an angle *θ*(*θ* = 1.5°(0.026rad))relative to joint *R*; each tooth *A*_*i*_ on horn shape blade curve RA_20_ is rotated to cut the material with reaming radius of OA*_i_*. The coordination of each tooth *A*_*i*_ on the curve RA_20_ is thus:

(4) $$\begin{array}{c} \left\{\begin{array}{c} x_{i}^{\prime \prime }=\left(x_{i}^{\prime }-36.6\right)\cdot \cos\left(\frac{j\cdot \theta \cdot \pi }{180}\right)-\left(y_{i}^{\prime }-86.3\right)\cdot \sin\left(\frac{j\cdot \theta \cdot \pi }{180}\right)+95,\\ y_{i}^{\prime \prime }=\left(x_{i}^{\prime }-36.6\right)\cdot \sin\left(\frac{j\cdot \theta \cdot \pi }{180}\right)+\left(y_{i}^{\prime }-86.3\right)\cdot \cos\left(\frac{j\cdot \theta \cdot \pi }{180}\right), \end{array}\right.\left(j=1,2,3,\cdots ,40\right). \end{array}$$

The reaming radius of each tooth on *A*_*i*_ (*i* = 1, 2, ⋯, 20) relative to the step unfolding rotation angle (*j*,*θ*) is then obtained with Equation (4) as:

(5) $$\begin{array}{c} r_{i}^{\prime }=\sqrt{{\left(x_{i}^{"}\right)}^{2}+{\left(y_{i}^{"}\right)}^{2}}. \end{array}$$

And the max cutting radius of tooth *A*_*i*_ at each step unfolding rotation angle (*j*,*θ*) is obtained as:

(6) $$\begin{array}{c} r_{j}=\max\left(r_{i}^{\prime }\right),\;j=1,2,\cdots ,40. \end{array}$$

The max reaming radius of the horn shape blade curve on each step unfolding rotation angle (*j*, *θ*) is taken as the varying reaming radius *r*_*j*_, it is shown with the related cutting tooth number in Figure 2(b).

As the unfolding rotation of the horn shape blade curve, teeth from *A*_15_ to *A*_20_ have behaved as the instantaneous max reaming radius tooth one by one, thus the reaming load has been sustained on those teeth range, which takes about 30% of all teeth, and the tooth *A*_20_ has even taken about 55% of the reaming cut, whereas the majority teeth from *A*_1_ to *A*_14_ have not conducted the reaming cut at all; for the horn shape blade curve, the reaming load is mostly distributed at the minority teeth from *A*_15_ to *A*_20_, and mostly the tooth *A*_20_; thus, serious wear and tear of the teeth will reduce the service life of the reamer bit. It is necessary to improve the utilization rate of teeth and reduce the reaming load concentration. Curve of the blade directly determines the relationship between reaming radius and tooth, thus the utilization rate of teeth and reaming load distribution. Inspired by the horn shape blade curve, a bionic blade curve was proposed.

### 3.2. Bionic Blade Curve

**Figure 3:**
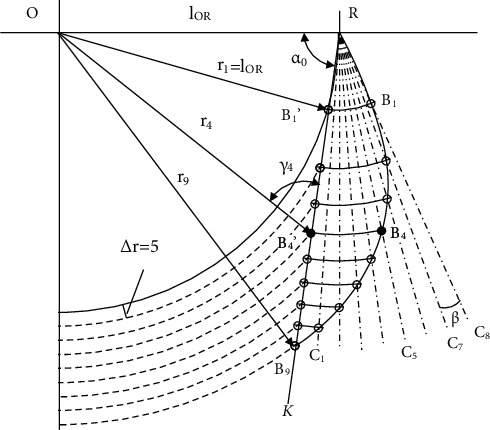
Bionic blade curve.

To improve the utilization rate and reduce the reaming load concentration of the tooth on the blade, the lateral reaming was designed to be started from the proximal tooth to the distal. The curve of the bionic blade was defined as following steps and shown in Figure 3. *B*_*i*_(*i* = 1, 2, ⋯, 9) is the typical point on the bionic blade curve *RB*_*i*_, the tip of teeth *T*_*i*_ is welded and overlap with point *B*_*i*_.

Step 1: an auxiliary line RK is defined through the rotation joint *R* on the bit body with an angle *α*_0_ relative to the minus of OR. A series of concentric arcs with radius of *r*_*i*_(*r*_*i*+1_ = *r*_*i*_ + 5) are intersected with line RK at points *B*_*i*_′; *B*_*i*_′ is the reference reaming point of each radius *r*_*i*_

Step 2: at the beginning of the anticlockwise unfolding rotation at joint *R* for the bionic blade curve *RB*_*i*_, the tooth *T*_1_ on point *B*_1_ is the first one to be rotated out and to ream the material, with the reaming point *B*_1_′ on line RK and radius *r*_1_. The reaming radius *r*_*i*_ of other teeth *T*_*i*_ on *B*_*i*_ (*i* ≠ 1) is shorter than *r*_1_; the reaming around radius *r*_1_ is done by the tooth *T*_*i*_

Step 3: the bionic blade curve *RB*_*i*_ is then further rotated with an angle ∠*KRC*_1_ (∠*KRC*_1_ = ∠*C*_*i*_*RC*_*i*−1_ = *β*, (*i* = 1, 2, ⋯, 9)), the tooth *T*_2_ on point *B*_2_ is rotated to the position *B*_2_′ on the line *RK*, the reaming radius is increased to *r*_2_. Tooth *T*_1_ on the position *B*_1_ is rotated to the radial line *RC*_1_, the reaming radius *r*_*i*_ of other teeth *T*_*i*_ (*i* ≠ 2) is shorter than *r*_2_, and the reaming cut around radius *r*_2_ is done by tooth *T*_2_

Step 4: the bionic blade curve *RB*_*i*_ is unfolded step by step with an angle∠*KRC*_1_, each tooth *T*_*i*_ on point *B*_*i*_ is rotated to the position of *B*_*i*_′ on line *RK*, and the related reaming radius becomes *r*_*i*_. *B*_*i*−1_ is rotated to the radial line *RC*_9+(1 − *i*)_, the reaming radius *r*_*j*_ of other tooth *T*_*j*_ on *B*_*j*_ (*j* ≠ *i*) is shorter than *r*_*i*_, and the reaming cut around radius *r*_*i*_ is done by tooth *T*_*i*_

Step 5: as the last point *B*_9_ is rotated to the line *RK*, the bionic blade curve *RB*_*i*_ is defined by the points *B*_*i*_(*i* = 1, 2, ⋯, 9). The position of each *B*_*i*_ can be obtained with the geometric relations from above steps.

Take point *B*_4_ as an example; in the triangle Δ*ORB*_4_′, it has:

(7) $$\begin{array}{c} \frac{{\text{l}}_{\text{OR}}}{\sin\gamma_{4}}=\frac{r_{4}}{\sin\alpha_{0}}. \end{array}$$

It deduces:

(8) $$\begin{array}{c} \gamma_{4}=\text{asin}\frac{{\text{l}}_{\text{OR}}\cdot \sin\alpha_{0}}{r_{4}}. \end{array}$$

For *l*_*RB*_4_′_ of *RB*_4_′; in the triangle Δ*ORB*_4_′, it also has:

(9) $$\begin{array}{c} \frac{{\text{l}}_{{{\text{RB}}_{4}}^{\text{'}}}}{\sin\left(\pi -\alpha_{0}‐\gamma_{4}\right)}=\frac{r_{4}}{\sin\alpha_{0}}. \end{array}$$

Thus,

(10) $$\begin{array}{c} l_{{RB}_{4}}=l_{{{RB}_{4}}^{\text{'}}}=\frac{r_{4}\cdot \sin\left(\pi -\alpha_{0}-\gamma_{4}\right)}{\sin\alpha_{0}}. \end{array}$$

With the *l*_*RB*_4__ and ∠*ORC*_5_ = *π* + *π* · (*α*_0_ + (9 − 4) · *β*)/180, the present coordination of B_4_ are:

(11) $$\begin{array}{c} \left\{\begin{array}{c} x_{B_{i}}=l_{{RB}_{4}}\cdot \cos\left(\pi +\pi \cdot \left(\alpha_{0}+\left(N-i\right)\cdot \beta \right)/180\right)+l_{\text{OR}},\\ y_{B_{i}}=l_{{RB}_{4}}\cdot \sin\left(\pi +\pi \cdot \left(\alpha_{0}+\left(N-i\right)\cdot \beta \right)/180\right), \end{array}\right.\;N=9,i=4. \end{array}$$

The position of each point *B*_*i*_ (*i* = 1, 2, ⋯, 9) on the bionic blade curve *RB*_*i*_ in Figure 3 can be obtained with Equation (11), and the shape of the bionic blade curve is thus determined. Before the normal lateral reaming, the bionic blade curve *RB*_*i*_ is firstly rotated clock-wise with an angle of (*N* − 1) · *β* to withdraw it inside the initial radius of *r*_1_. The coordination of each point *B*_*i*_ can be further deduced when the bionic blade is unfolded again with a smaller step angle of *β*/2 in the normal reaming process as:

(12) $$\begin{array}{c} \left\{\begin{array}{c} x_{B_{ij}}^{\text{'}}=l_{{RB}_{i}}\cdot \cos\left(\pi +\pi \cdot \left(\alpha_{0}-\left(N-1\right)\cdot \beta +\left(N-i\right)\cdot \beta +j\cdot \beta /2\right)/180\right)+l_{\text{OR}},\\ y_{B_{ij}}^{\text{'}}=l_{{RB}_{i}}\cdot \sin\left(\pi +\pi \cdot \left(\alpha_{0}-\left(N-1\right)\cdot \beta +\left(N-i\right)\cdot \beta +j\cdot \beta /2\right)/180\right). \end{array}\right. \end{array}$$

Here, *N* = 9, *i* = 1, 2, ⋯, 9, *j* = 1, 2, ⋯, 16; *j* is the serial number of step unfolding rotation for the bionic blade; the reaming radius of each tooth *T*_*i*_ on *B*_*i*_ () relative to the step rotation is thus:

(13) $$\begin{array}{c} r_{{cT}_{ij}}=\sqrt{{\left(x_{B_{ij}}^{\text{'}}\right)}^{2}+{\left(y_{B_{ij}}^{\text{'}}\right)}^{2}}. \end{array}$$

And the max radius of the bionic blade curve on each step unfolding rotation is obtained as:

(14) $$\begin{array}{c} r_{j}\left(\alpha_{0},\beta \right)=\max r_{{cT}_{ij}},i=1,2\cdots N,j=1,2...16. \end{array}$$

**Figure 4:**
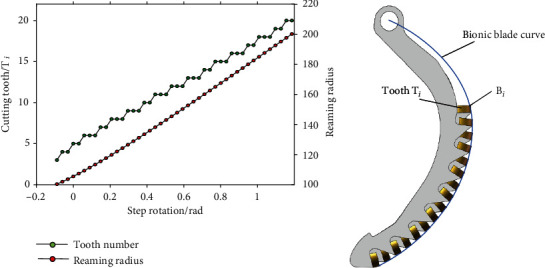
Cutting characteristic of bionic blade. (a) Reaming radius and related tooth. (b) Bionic blade curve and actual structure.

The parameters for the bionic blade curve *RB*_*i*_ can be further increased to *N* = 20, *j* = 40, *r*_1_ = 100, *r*_20_ = 200; the reaming radius can be changed from 100 mm to 200 mm. The instantaneous max reaming radius *r*_*j*_ of each step rotation is related to a tooth *T*_*i*_, and the relation between *r*_*j*_ and *T*_*i*_ is affected by the angles *α*_0_ and *β*. The preferred parameters of them were determined as *α*_0_ = 88° and *β* = 3.4° finally to decentralize the cutting load to majority of teeth. And the reaming radius *r*_*j*_ and related tooth number *T*_*i*_ were calculated with Equations (13)–(14) and shown in Figure 4(a). The bionic blade reams from tooth *T*_3_ to *T*_20_ as it is rotated; it means 90% of the teeth are functional in the reaming process, and each tooth *T*_*i*_ cuts about two or three step rotations of *β*/2. The actual simplified bionic blade curve and structure in this study are shown in Figure 4(b); 16 teeth were assembled row by row as a group on the bionic blade, to ream the rock hole from 220 mm to 400 mm in diameter. The back angle of each tooth *T*_*i*_ was set as 12 degrees relative to the curve normal at point *B*_*i*_ to work with a low reaming load [20].

### 3.3. Compare of Two Blades

As is shown in Table 1, both the horn shape blade and bionic blade are arranged 20 teeth on them to cut the material. The teeth from the 15^th^ to the 20^th^ on the horn shape blade could cut the material with a utilization rate of 30%, and the teeth from 3^rd^ to the 20^th^ on the bionic blade can conduct the reaming cut with the utilization rate of 90%. The reaming load concentration of the 30% teeth (especially for the 20^th^ tooth) on the horn shape blade will cause unexpected wear and tear, thus reducing the bit life. As the reaming load can be averagely and linearly decentralized to 90% of the teeth on the bionic blade, it is more efficient and economical to use the bionic blade curve in the lateral reaming procedure.

### 3.4. Mechanical Structure of the Bionic Lateral PDC Reamer Bit

**Figure 5:**
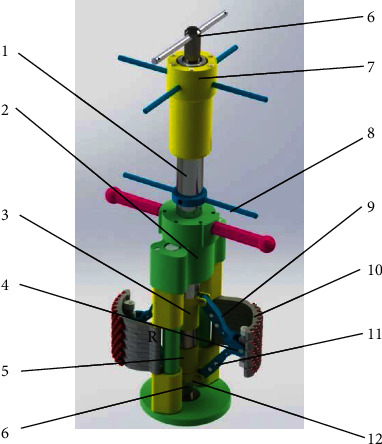
Mechanical structure. 1: Push rod; 2: main body; 3: upper slider; 4: universal joint pin; 5: lower slider; 6: guide column; 7: drive handle; 8: fixed handle; 9: upper push bar; 10: bionic blade; 11: lower push bar; 12: pull rod.

To ream the hole inside the rock material, a straight hole of small diameter should be drilled first, and later, the small hole can be reamed laterally. The first step can be done by the existing PDC bit without difficulty; the second step is the key work in this research with the bionic lateral PDC reamer bit, as the mechanical structure is shown in Figure 5. Two symmetrical bionic blades (shown in Figure 4(b)) were installed inside the main body via rotation joints *R* shown in Figure 5; the PDC teeth were welded on the external surface of the bionic blades to ream the side material of the small inner hole. The bionic blade was drove by the upper push bar and lower push bar, which were further motivated by the drive handle and the fixed handle outside the bit body with a screw transmission.

## 4. Simulation and Experiment

### 4.1. Simulation of the Bionic Lateral PDC Reamer Bit

**Figure 6:**
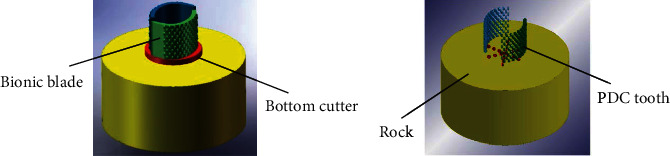
Simplified model of rock, bottom cutter, and bionic blade. (a) Model with rock, bottom cutter, bionic blade, and PDC teeth. (b) Model with only rock and PDC teeth.

The whole working process of the bionic lateral PDC reamer bit should include two parts: bottom cutter drilling and lateral reaming. A small-diameter vertical inner hole is expected to be drilled into the rock firstly by the bottom cutter, latter the inner hole is reamed by the bionic lateral PDC reamer bit. This article focuses on the second process of the bionic reaming, as the research about drill a vertical hole is relatively common [21]. The simplified models of the bottom cutter, bionic blade, PDC teeth, and rock were established in SolidWorks, as shown in Figure 6(a), and then imported into ANSYS/LS-DYNA for simulation [22, 23]. The rock was a cylinder shape with a diameter of 450 mm and a height of 200 mm. The drilling diameter of the bottom cutter was 220 mm; the height of the bionic blade reamer bit was designed as 130 mm to reduce the computational cost. The body of the bottom cutter and bionic blade was further concealed, as shown in Figure 6(b), since the material cutting occurs only between the PDC teeth and rock. The most common shape of PDC tooth is cylindrical, despite of developed and used conical, ridged diamond, axe shapes of teeth [24, 25]. The mechanism of rock-breaking for special-shaped PDC teeth such as conical PDC tooth etc. is not clear so far in theory and needs further investigation. As the most common shape of PDC tooth, the cylindrical PDC tooth was chosen for the manuscript; special-shaped PDC teeth such as conical PDC tooth etc. were not considered in the current research. Each PDC tooth on the bottom cutter and bionic blade was cylindrical shape with a diameter of 13 mm and thickness of 5 mm. The angle between the normal of the rock surface and the cutting surface of the tooth was referred as the back rack angle. The back rake angle was set as 12°, which is considered to achieve a low resistance force in rock cutting [26, 27].

**Figure 7:**
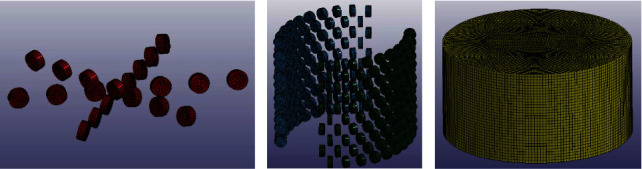
(a) PDC teeth on bottom cutter. (b) PDC teeth on bionic blades. (c) Rock.

In the simulation, as the hardness (10 with Mohs hardness scale) and strength (about 1500 MPa) of PDC teeth are much higher than those of the rock (3 with Mohs hardness scale and 60 MPa), the wear of PDC teeth in rock-breaking process is ignored. In order to improve calculation efficiency, like the other researches commonly did [28, 29], the PDC teeth were regarded as rigid bodies and divided into a tetrahedral free mesh, the mesh size was 1 mm, and the parameters were set as those of common carbon steel and given in Table 2. The type of the rock used in the simulation is limestone, which is widely distributed around the world. Quantity of researches on limestone has been done; the material parameters of the limestone are easy to obtain, and it has little difficulty in replicating and verifying the simulation result. The surface of rock was considered smooth, ignoring the effect of environmental factors, such as temperature. The rock model was a hexahedral element; the mesh size was 5 mm. RHT constitutive model was selected for the rock to well simulate rock's mechanical properties, and the main parameters of the rock model are given in Table 3. The meshing models are shown in Figure 7. As the bionic lateral PDC reamer bit breaks the rock, it breaks through the surface of the rock and makes contact with the rock interior. The contact type was surface-to-surface eroding contact.

In the first vertical inner hole drilling process, the bottom cutter PDC teeth break down the rock under the resultant action of feeding and revolution. As the feeding motion continues, the rock damage continuously moves vertically, and the inner hole is formed with the depth being increased. In the second lateral reaming process, the breaking of rock is basically similar to that in the first drilling process. The difference is that the cutting surface changes from a horizontal plane to a vertical cylindrical surface. The overall process of rock fragmentation with the bionic lateral PDC reamer bit is illustrated in Table 4. The vertical inner hole in the rock was first drilled out with a depth of approximately 192 mm and a diameter of approximately 220 mm, from 0 to about 250 ms. The inner hole was then reamed by the PDC teeth on the bionic blades from 250 to 570 ms, and the middle position of the inner hole was reamed to a diameter of approximately 396 mm and a height of approximately 137 mm.

**Figure 8:**
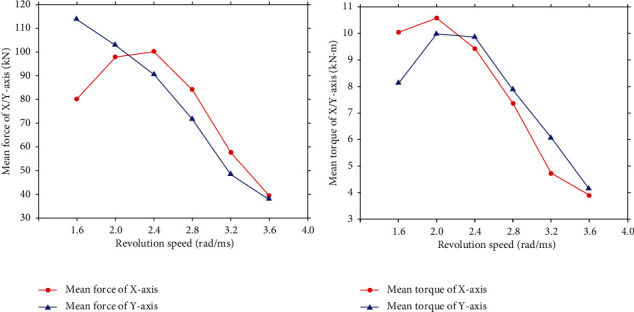
The impact of revolution speeds on mean force and torque on the PDC teeth of bionic blade. (a) Mean force. (b) Mean torque.

Multigroup simulations were further conducted firstly with the revolution speed being varied in the second process to study the effect of it to the reaming load. In these simulations, the rotation feed speed of bionic blade was kept as 3.5 × 10^−3^ rad/ms while the revolution speed was varied as 1.6, 2.0, 2.4, 2.8, 3.2, and 3.6 rad/ms. The mean force and torque acting on the PDC teeth on the bionic blades at different revolution speeds were obtained and shown in Figure 8. The curves thus show the load acting on PDC teeth decreases with an increase of revolution speed. The result is reasonable as the higher revolution speed reduces the volume of rock cut by the PDC teeth within a cutting circle when the rotation feed speed is fixed.

**Figure 9:**
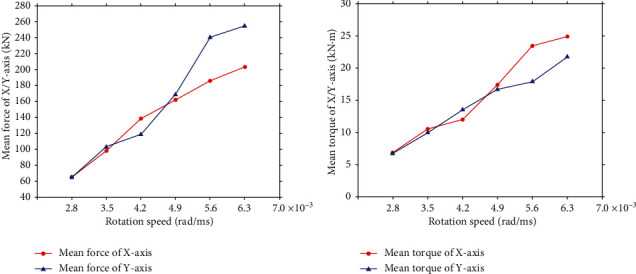
The impact of rotation speeds on mean force and torque on the PDC teeth of bionic blade. (a) Mean force. (b) Mean torque.

Adopting the same method, the rotation feed speed of the bionic blade for the reaming process was changed as 2.8, 3.5, 4.2, 4.9, 5.6, and 6.3 × 10^−3^ rad/ms for the multigroup simulation with the revolution speed was kept as 2 rad/ms. The mean force and torque acting on the PDC teeth of the bionic blades at different rotation speeds are shown in Figure 9. The curves in the figure again show the load acting on the PDC teeth increases as the rotation speed increases. The simulation results are acceptable as an increase in rotation speed directly enlarges the rock volume for the PDC teeth to cut which leads to an increase in the load acting [22, 30, 31].

### 4.2. Prototype and Indoor Experiment

**Figure 10:**
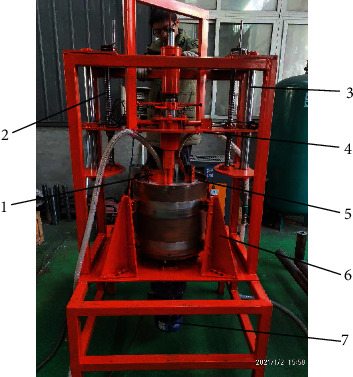
Experiment setup for bionic lateral PDC reamer bit. 1: bionic blade; 2: lift screw; 3: guide rail; 4: lift board; 5: concrete sample; 6: side supporter; 7: motor.

The prototype of the bionic lateral PDC reamer bit has been built as shown in Figure 10, and the lateral reaming test was carried out with it indoors. The max reaming dimension was designed as 400 mm in diameter and 130 mm in height one time to ease the test procedure. The main body of the bionic lateral PDC reamer bit was arranged to be still, and the reaming target sample was drove to be rotated by motor relative to the bit axis. The reaming sample was made of concrete embedded with small rocks around 10 mm in dimension, as the standard cylindrical rock sample of 450 mm in diameter was difficult to get. As mentioned above, this study put emphasis on the reaming process; the concrete sample was constructed as a tubular shape with an inner hole of 240 mm in diameter, which is larger than the initial reaming diameter 220 mm of the prototype bit to ease the preparation of test. The concrete sample was rotationally installed under the bionic lateral PDC reamer bit, four side supporters were arranged around it to improve the rotation precision of the sample. The bionic lateral PDC reamer bit was installed on a lifting board, which was drove by screw mechanism and guided with the guide rail, to land the bionic lateral PDC reamer bit to the inner hole of the concrete sample. A pump circulation system was employed to provide water flow to cool the reaming PDC teeth and take away the drill cuttings made inside the concrete sample.

**Figure 11:**
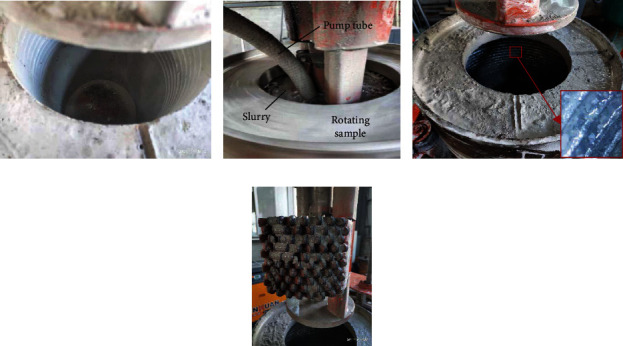
(a) Sample before reaming. (b) Reaming the sample. (c) Inside of reamed sample. (d) Bionic blade after reaming.

Concrete samples were reamed in the indoor test, and the reaming result of the concrete sample is shown in Figure 11. The concrete sample is shown in Figure 11(a). The inner hole of the concrete sample was finally reamed from 240 mm to 407 mm in diameter, as shown in Figures 11(b) and 11(c). The surface of the reamed hole was cut with corrugate shape, as shown in Figure 11(c); it was made by the serried teeth rows on the bionic blades. The corrugate surface of the reamed hole indicated that the small rocks in the concrete sample were tightly fixed without breakage during the reaming process, which was considered to be qualified for the reaming validation. During the reaming process of the concrete sample, no slag had been generated by the bit; all the materials in the cutting area was transformed to fines and then mixed with water into slurry, as shown in Figure 11(b). The transmission mechanism, bionic blades, and PDC teeth were in good condition after reaming the concrete sample; no macroscopic wear was found at the cutting edge of the teeth. As the flow magnitude of the pump was not powerful enough, the bit balling was found on the bionic blades, as is shown in Figure 11(d), which should be improved in the future.

## 5. Conclusions

1. To dig and expand the hole in the rocky areas, a lateral reamer bit which integrated the bionics mechanism and PDC bit technology was proposed in this study. The shape and structure of the *Capra sibirica* horn was utilized for designing the blade of the reamer bit to control the reaming radius
2. The horn shape blade curve for the lateral reamer bit was obtained and evaluated, found with an utilization rate of 30% of the total teeth, and also the load concentration of the last PDC tooth. A bionic blade curve evolved from the horn shape was defined with geometric method mainly considering the angle *α*_0_ of the auxiliary line RK and the step rotation angle *β*, with a utilization rate of 90% of the teeth obtained. Mechanical structure of the bionic lateral PDC reamer bit was designed with two symmetrical lateral bionic blades drove by the screw mechanism
3. Multigroup simulations were conducted; the effects of the revolution speed and rotation feed speed of the bionic blade to force and torque acting on the PDC teeth were studied. The force and torque of PDC teeth on the bionic blades decreases with the increasing of revolution speed, but increases as the rotation feed speed increases. The simulation results of the bionic lateral PDC reamer bit were in accordance with the established bit knowledge
4. Concrete samples with tubular shape were employed to test the bionic lateral PDC reamer bit indoors. The inner holes of the concrete samples were reamed from 240 mm to 407 mm in diameter, with the PDC teething on the bionic blades behaving as expected. The test results approved the bionic design of the lateral PDC reamer bit, and the lateral reaming was achieved in the rock material

## Figures and Tables

**Table 1:** Compare of the blades.

Name	Rotation angle variance of the teeth	First reaming tooth number	Last reaming tooth number	Number of the teeth for cutting	Utilization rate of the teeth
Horn shape blade	0.016	15	20	6	30%
Bionic blade	0.00081	3	20	18	90%

**Table 2:** Material parameters of the PDC teeth.

Density (kg/m^3^)	Young's modulus (GPa)	Poisson's ratio
7800	200	0.3

**Table 3:** Material parameters of limestone RHT model.

RO (kg/mm^3^)	Shear (GPa)	EPSF (GPa)	B0	B1	T1 (GPa)	*A*	*N*
2.66 × 10^−6^	20.529	0.3	1.22	1.22	35.27	1.6	0.61
FC (GPa)	FS^∗^	FT^∗^	Q0	B	T2 (GPa)	E0C (ms^−1^)	E0T (ms^−1^)
0.11	0.18	0.1	0.6805	0.0105	0	3.00 × 10^−8^	3.00 × 10^−9^
EC (ms^−1^)	ET (ms^−1^)	BETAC	BETAT	PTF	GC^∗^	GT^∗^	XI
1.00 × 1027	1.00 × 1027	1.14 × 10^−2^	1.54 × 10^−2^	0.001	0.53	0.7	0.5
D1	D2	EPM	AF	NF	Gamma	A1 (GPa)	A2 (GPa)
0.04	1	0.01	1.6	0.61	0	35.27	39.58
A3 (GPa)	PEL (GPa)	PCO (GPa)	NP	Alpha			
9.04	0.0733	6	3	1.119			

**Table 4:** Cutting process of the bionic lateral PDC reamer bit.

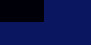	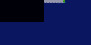	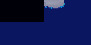	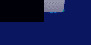	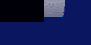	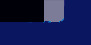	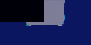
0 ms	20.6 ms	50.1 ms	85.5 ms	115.0 ms	147.5 ms	174.3 ms
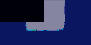	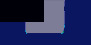	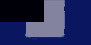	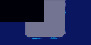	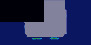	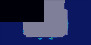	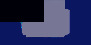
203.5 ms	227.1 ms	253.7 ms	282.1 ms	311.6 ms	340.1 ms	368.6 ms
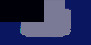	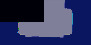	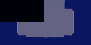	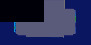	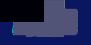	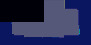	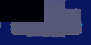
397.1 ms	425.6 ms	453.1 ms	482.6 ms	512.9 ms	541.5 ms	570.0 ms

## Data Availability

The data used to support the findings of this study are included within the article.
